# Trends in prostate cancer mortality in the state of São Paulo, 2000 to 2015

**DOI:** 10.11606/s1518-8787.2020054001948

**Published:** 2020-08-24

**Authors:** Carolina Terra de Moraes Luizaga, Karina Braga Ribeiro, Luiz Augusto Marcondes Fonseca, José Eluf

**Affiliations:** I Fundação Oncocentro de São Paulo Diretoria de Informação e Epidemiologia São PauloSP Brasil Fundação Oncocentro de São Paulo. Diretoria de Informação e Epidemiologia. São Paulo, SP, Brasil; II Faculdade de Ciências Médicas Santa Casa de São Paulo Departamento de Saúde Coletiva São PauloSP Brasil Faculdade de Ciências Médicas da Santa Casa de São Paulo. Departamento de Saúde Coletiva. São Paulo, SP, Brasil; III Universidade de São Paulo Faculdade de Medicina Hospital das Clínicas São PauloSP Brasil Universidade de São Paulo. Faculdade de Medicina. Hospital das Clínicas. Serviço de Imunologia Clínica e Alergia. São Paulo, SP, Brasil; IV Universidade de São Paulo Faculdade de Medicina Departamento de Medicina Preventiva São PauloSP Brasil Universidade de São Paulo. Faculdade de Medicina. Departamento de Medicina Preventiva. São Paulo, SP, Brasil; V Fundação Oncocentro de São Paulo São PauloSP Brasil Fundação Oncocentro de São Paulo. São Paulo, SP, Brasil

**Keywords:** Prostate neoplasms, mortality, Mortality, trends, Age distribution, Temporal distribution

## Abstract

**OBJECTIVE:**

To estimate the magnitude and identify patterns of change in prostate cancer mortality in the state of São Paulo and in the 17 regional health care networks, according to age groups from 50 years onwards, in the period between 2000 to 2015.

**METHODS:**

Age-adjusted mortality rates (per 100,000 men) were calculated by the direct method using the Segi world population as standard. Joinpoint regression was used to calculate the average annual percent change (AAPC), with a confidence interval of 95% (95%CI), by regional network and age group (50–59, 60–69, 70–79 and 80 years or more).

**RESULTS:**

For the state of São Paulo, age-adjusted mortality rates were 15.2, 13.3 and 11.9 per 100,000 men, respectively, in the periods between 2000 to 2005, 2006 to 2010 and 2011 to 2015, with a significant decrease trend (AAPC = -2.10%; 95%CI -2.42 – -1.79) each year. Among the 17 networks, 11 presented significant mean annual reductions, ranging from -1.72% to -3.05%. From the age of 50 onwards, there was a sharper reduction in the groups from 50 to 59 (AAPC = -2.33%; 95%CI -3.04 – -1.62) and 60 to 69 years (AAPC = -2.84%; 95%CI – 3.25 – -2.43).

**CONCLUSION:**

Although reductions in mortality are still slight, they indicate progress in prostate cancer control actions. Screening actions and changes in therapeutic behaviors in recent decades may be modifying incidence and survival, resulting in changes in the mortality profile. More detailed studies will be useful in understanding the factors that lead to the interregional variations found.

## INTRODUCTION

Prostate cancer is the second most incident and the sixth leading cause of cancer death in men worldwide^1^, but trends in incidence and mortality from the disease vary in several countries. Geographic variations in incidence rates are probably due to the combination of underlying prevalent cases and differences in screening-related practices, including prostate-specific antigen (PSA) examination^2,3^.

Brazil is a large country with regional disparities, resulting in different patterns of occurrence of diseases, including cancer. It is estimated that, in the country, prostate cancer is the most common type of cancer in men, with an expected number of 68,220 new cases of the disease in 2018 and 2019. This number corresponds to 31.7% of all cancers (except non-melanoma skin cancer) and to gross and age-adjusted incidence rates of 66.1 and 66.8 new cases/100,000 men, respectively. For the state of São Paulo, in the same year, 14,890 new cases were estimated as well as an age-adjusted incidence rate of 59.8 new cases/100,000 men^4^. The prostate cancer age-adjusted mortality rate in Brazil, from 2006 to 2010, was 13.7/100,000 men, with a projection of 12.9/100,000 for the period 2011 to 2015. For the Southeast region, these rates were 12.9 and 11.3/100,000 in the respective periods^5^.

Regarding cancer treatment by the Unified Health System (SUS), there is evidence that access to diagnosis and treatment is still heterogeneously distributed in São Paulo^6^, suggesting distinct patterns in cancer mortality. This study aimed to estimate magnitude and identify patterns of change in prostate cancer mortality both in general and according to age groups from 50 years onwards, in the state of São Paulo and in each of its 17 regional health care networks (RHCN), in the period between 2000 and 2015.

## METHODS

This is an ecological study, which analyzed the data series from 2000 to 2015. Deaths from prostate cancer constituted the object of study. We selected the cases that occurred among residents of the 17 health networks in the state of São Paulo. The division of the territory into 17 health networks, made in 2012, aimed to guarantee the universality and integrality of health care to the entire population of São Paulo. RHCN are defined as organizational arrangements of health services to integrate actions and organize systems and information flows, supporting planning and setting of dynamics in the territory^7^.

Data on deaths were collected on the website of the Department of Informatics of the Unified Health System (Datasus), by downloading the files from the Mortality Information System of the Ministry of Health^8^. The variables selected were age group, place of residence, year and underlying cause of death classified with code C61 of the 10th revision of the International Classification of Diseases^9^. The male population in the state of São Paulo was obtained by tabulating information on population estimates^10^ for the period between 2000 and 2015 on the Datasus website. To make the spatial representation of mortality rates in the last period of the series, the Tab software for Windows (Tabwin version 3.6 b) was used^11^.

Deaths were aggregated into groups within five-year intervals (from 0 to 80 years or more) and ten-year intervals (50 to 80 years or more). We calculated age-adjusted mortality rates based on 100,000 men. Age adjustment was made by the direct method, using as standard the age composition of the Segi world population^12^. To represent magnitude of mortality, the rates were calculated for three-year periods between 2000 and 2015: period 1 (2000–2005), period 2 (2006–2010) and period 3 (2011–2015). For analysis of temporal trend, we calculate the rates for each year of the series and for four age groups (50–59, 60–69, 70–79, and 80 years or older).

The evaluation of the temporal trend was performed by Joinpoint regression to identify the points of change in a period and to verify the statistical significance of trends in age-adjusted mortality rates^13^. We estimated the synthesis measures of trend analysis, otherwise known as average annual percentage change (AAPC)^14^, which indicated the increase or decrease and magnitude of the changes in the period from 2000 to 2015 and in age groups from 50 years, accompanied by the respective confidence intervals of 95% (95%CI), with the use of the software Joinpoint Regression, version 4.2.0.115. We maintained the software’s default option for adjusting an uncorrelated error model after tests for serial autocorrelation indicated that the analysis was safe from misinterpretation.

As this is an ecological study, data collection was performed in public domain databases, with no need to submit the project to a research ethics committee.

## RESULTS

From 2000 to 2015, there were 40,631 deaths from prostate cancer among men residing in the state of São Paulo (Table 1). In the first period (2000–2005), the age-adjusted mortality rate was 15.2 deaths/100,000 men. Among regional health networks, rates ranged from 12.1/100,000 (RHCN 12) to 18.3/100,000 (RHCN 4). In subsequent periods, the rates for the state were 13.3 and 11.9/100,000 in 2006–2010 and 2011–2015, respectively. In the last period, the highest rates were observed in RHCN 3 and 5 (15.1 and 14.6/100,000) and the lowest in RHCN 10 and 12 (9.3/100 thousand).

**Table 1:** Regional health care networks (RHCN) according to regional health department (RHD) and corresponding health regions, residing male population in 2015, number of deaths and age-adjusted mortality rates of prostate cancer between 2000 and 2015.

RHCN	RHD (region)	Residing male population (2015)	Number of deaths (2000–2015)	Age-adjusted mortality rates (per 100,000 men)		
				2000–2005	2006–2010	2011–2015
1	Greater São Paulo (Greater ABC)	1,327,156	2.263	15.7	13.1	12.7
2	Greater São Paulo (Guarulhos and Alto do Tietê)	1,431,263	1.908	16.2	14.8	14.0
3	Greater São Paulo (Franco da Rocha)	290,202	354	17.4	14.8	15.1
4	Greater São Paulo (Mananciais)	539,967	635	18.3	14.4	13.3
5	Greater São Paulo (Rota dos Bandeirantes)	898,382	1.241	17.7	15.4	14.6
6	Greater São Paulo (City of São Paulo)	5,739,347	11.658	16.8	14.7	12.2
7	Baixada Santista and Registro	1,012,305	2.347	15.1	15.5	13.9
8	Sorocaba	1,225,859	2.144	14.2	13.2	11.5
9	Bauru	878,978	1.852	14.2	12.3	11.4
10	Marília	566,350	1.245	13.6	11.1	9.3
11	Presidente Prudente	385,065	861	12.4	12.0	10.3
12	Araçatuba São José do Rio Preto	1,180,992	2.346	12.1	10.0	9.3
13	Araraquara, Barretos, Franca and Ribeirão Preto	1,780,446	3.591	14.4	13.5	11.9
14	Piracicaba	764,711	1.386	13.5	11.6	11.5
15	Campinas and São João da Boa Vista (Rio Pardo, Mantiqueira, Baixa Mogiana, Oeste VII, Campinas)	2,005,472	3.543	15.1	11.8	11.1
16	Campinas (Bragança and Jundiaí)	612,182	1.136	15.4	12.3	11.4
17	Taubaté	1,222,358	2.121	14.1	13.7	13.2
**State of São Paulo**		**21,861,035**	**40,631**	**15.2**	**13.3**	**11.9**

Sources: population: demographic estimates by the Interagency Network for Health Information (RIPSA/Ministry of Health^10^); deaths: Mortality Information System/Ministry of Health (Datasus)^8^.

**Figure 1:**
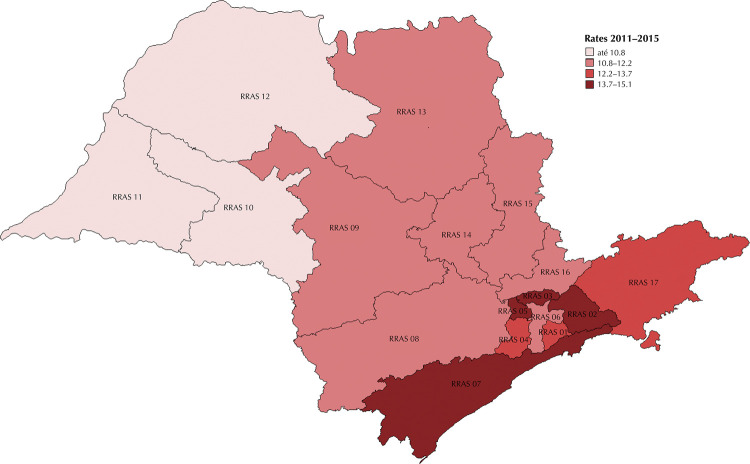
Age-adjusted mortality rates (per 100,000 men) for prostate cancer according to the 17 regional health care networks in the state of São Paulo from 2011 to 2015.

**Figure 2:**
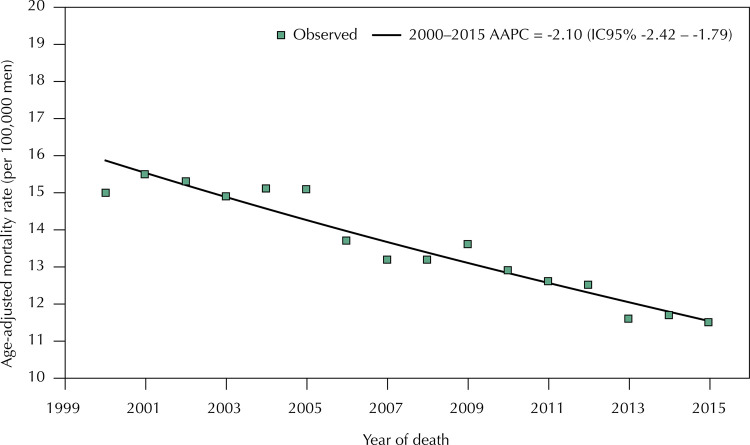
Trend of age-adjusted mortality rates due to prostate cancer in the state of São Paulo from 2000 to 2015.

In the period studied, there was a statistically significant decrease in prostate cancer mortality rates in the state of São Paulo (AAPC = -2.10%; 95%CI -2.42 – -1.79) (Figure 2). Regarding regional distribution, the same trend was observed in 11 of the 17 regions, with average annual reductions between -1.72% (95%CI -2.46 – -0.98), in RHCN 13, and -3.05% (95%CI -4.95 – -1.10), in RHCN 10. In the other regions, there was a trend of reduction without statistical significance.

**Table 2:** Trend of age-adjusted mortality rates for prostate cancer in the 17 regional health care networks (RHCN) from 2000 to 2015.

RHCN	AAPC	95%CI
1	**-2.08**	-2.93 – -1.22
2	-1.05	-2.15 – 0.06
3	-1.27	-3.49 – 1.00
4	**-2.77**	-4.16 – -1.36
5	**-2.41**	-4.21 – -0.59
6	**-2.96**	-3.31 – -2.60
7	-0.60	-1.60 – 0.42
8	**-1.89**	-3.48 – -0.28
9	**-1.90**	-3.20 -0.58
10	**-3.05**	-4.95 – -1.10
11	-1.26	-3.02 – 0.52
12	**-2.44**	-3.21 – -1.66
13	**-1.72**	-2.46 – -0.98
14	-1.28	-2.91 – 0.37
15	**-2.75**	-3.77 – -1.72
16	**-2.63**	-4.15 – -1.07
17	-0.52	-1.51 – 0.49

AAPC: average annual percentage change; 95%CI: 95% confidence interval

Observation: AAPC statistically different from zero in bold

Between 2000 and 2015, 99% of prostate cancer deaths occurred from the age of 50. Age-specific mortality rates showed great variation as well as increased risk of death with advancing age. For the state of São Paulo, in the last year of the series, the mortality rates for the four ten-year groups between 50 and 80 years or more were, respectively, 5.5, 34.4, 152.5 and 408.8 deaths/100,000 men.

The trend analysis according to age groups indicated a statistically significant reduction in mortality in all groups from 50 years onwards in the state (Table 3), with the highest percentages of reduction in the ranges of 50 to 59 (AAPC = -2.33; 95%CI -3.04 – -1.62) and 60 to 69 years (AAPC = -2.84; 95%CI -3.25 – -2,43).

**Table 3:** Trend of age-adjusted mortality rates due to prostate cancer according to age groups in the 17 regional health care networks (RHCN) and in the state of São Paulo from 2000 to 2015.

RHCN	AAPC (95%CI)			
	50 to 59	60 to 69	70 to 79	80 years onwards
1	-1.10 (-4.56 – 2.48)	-2.23 (-4.99 – 0.62)	**-1.53 (-2.94 – -0.09)**	**-2.71 (-4.36 – -1.04)**
2	0.41 (-3.29 – 4.25)	**-4.03 (-6.85 – -1.12)**	0.29 (-1.46 – 2.07)	-0.91 (-2.92 – 1.15)
3	- *	**-5.90 (-9.62 – -2.03)**	**3.26 (0.19 – 6.41)**	-2.84 (-7.18 – 1.70)
4	- *	-3.98 (-9.30 – 1.65)	-2.79 (-7.33 – 1.97)	**-2.98 (-5.22 – -0.68)**
5	**-6.04 (-11.02 – -0.79)**	-2.67 (-5.60 – 0.34)	-1.79 (-4.08 – 0.54)	-2.33 (-4.62 – 0.02)
6	-1.92 (-4.05 – 0.25)	**-2.77 (-3.38 – -2.16)**	**-3.47 (-4.30 – -2.63)**	**-3.43 (-4.86 – -1.97)**
7	-1.58 (-7.03 – 4.18)	**-1.75 (-3.33 – -0.15)**	-0.68 (-2.21 – 0.88)	0.30 (-1.62 – 2.26)
8	1.16 (-15.42 – 20.98)	1.12 (-8.11 – 11.27)	-0.75 (-2.17 – 0.70)	-1.87 (-4.03 – 0.35)
9	-0.59 (-5.70 ;4.80)	**-2.77 (-5.05 – -0.44)**	**-1.88 (-3.59 – -0.15)**	-1.82 (-3.65 – 0.05)
10	- ^a^	**-4.44 (-7.09 – -1.72)**	-2.47 (-5.16 – 0.30)	**-2.55 (-4.75 – -0.29)**
11	- ^a^	**-3.07 (-6.04 – -0.01)**	-1.50 (-3.76 – 0.80)	0.32 (-2.04 – 2.73)
12	-2.52 (-7.23 – 2.43)	**-2.40 (-3.29 – -1.50)**	**-3.24 (-4.31 – -2.16)**	-1.42 (-3.01 – 0.19)
13	-0.51 (-3.20 – 2.25)	**-3.65 (-5.23 – -2.05)**	**-0.99 (-1.96 – -0.01)**	**-1.58 (-2.94 – -0.20)**
14	- *	-1.30 (-4.31 – 1.81)	-1.18 (-3.35 – 1.03)	-1.83 (-4.38 – 0.79)
15	**-4.79 (-6.99 – -2.55)**	**-3.44 (-5.23 – -1.61)**	**-2.02 (-3.40 – -0.62)**	**-2.74 (-4.14 – -1.32)**
16	-4.40 (-8.61 – 0.00)	-2.18 (-5.00 – 0.73)	-2.12 (-4.64 – 0.47)	**-2.95 (-4.79 – -1.08)**
17	-4.41 (-9.08 – 0.49)	**-3.93 (-6.15 – -1.65)**	0.26 (-1.30 – 1.84)	0.57 (-1.55 – 2.73)
ESP	**-2.33 (-3.04 – -1.62)**	**-2.84 (-3.25 – -2.43)**	**-1.93 (-2.36 – -1.50)**	**-1.92 (-2.40 – -1.44)**

AAPC: average annual percentage change; 95%CI: 95% confidence interval

Observation: AAPC statistically different from zero in bold

^*^AAPC was not calculated as there was no record of death from prostate cancer in at least one year of the series between 2000 and 2015.

In the analysis stratified by regions, in the group of 50 to 59 years, significant reductions were observed in RHCN 5 (AAPC = -6.04; 95%CI -11.02 – -0.79) and RHCN 15 (AAPC = -4.79; 95%CI -6.99 – -2,55). In the group aged 60 to 69 years, in 11 of the 17 RHCN, there were significant reductions in mortality, especially in RHCN 2 and RHCN 3. In the group aged 70 to 79 years, the greatest decline (AAPC = -3.47; 95%CI -4.30 – -2.63) was observed in RHCN 6 and significant reductions of at least 0.99% in five other RHCN. In the range of 80 years or more, significant decreases were observed in seven regions, and RHCN 6 presented the highest magnitude reduction (AAPC = -3.43; 95%CI -4.86 – -1.97).

## DISCUSSION

In the period from 2000 to 2015, there was a statistically significant decrease in prostate cancer mortality rates in the state of São Paulo and in 11 of the 17 RHCN. Worldwide, the temporal trend of reducing mortality from this type of cancer was found in developed areas such as the United Kingdom (-1.14% each year between 1992 and 2004) and the United States (-4.17% between 1994 and 2004^16^ and -7.19% between 2009 and 2013)^17^. For Brazil and its regions, between 1980 and 2010, an upward trend was observed in a study that included the redistribution of deaths due to ill-defined causes in the correction of mortality rates^18^.

In the state of São Paulo, from 2011 to 2015, the age-adjusted mortality rate for prostate cancer (11.9 deaths/100,000 men) was lower than the estimated risk of death for South America in 2018^1^ (14/100,000), higher than estimated for North America, Southern Europe and Western Asia (8/100,000), and similar to the one estimated for the United Kingdom (13/100,000), Colombia and Argentina (12/100,000)^1^. Globally, prostate cancer mortality rates have lower geographic variability than the incidence of the disease^1^. The greatest risks of death are observed in less developed regions where there is a predominance of black population, such as in the Caribbean and Sub-Saharan Africa (rates from 23 to 27/100,000)^1^. As for the temporal trend, declines were previously noted in areas with more resources, while increases occurred in countries with few^2^.

Mortality studies are essential to indicate public health priorities; however, interpretations from this isolated measure may be fallacious. Trends in cancer mortality are the result of previous trends in both incidence and survival^19^. In both the state of São Paulo and Brazil as a whole, the data for incidence of cancer is available to a few municipalities covered by population-based cancer registries (RCBP), or is produced through estimates for states and capitals by the National Cancer Institute since 1995^4^. For methodological reasons, these estimates should not be used for time-series studies. Although there are five cancer registries in operation in the state, each providing incidence data for their municipalities^20^, it was not possible to evaluate the effects of incidence rates and survival of prostate cancer on mortality from the disease, either by the small population representation at the state level, or by instability in rates over time.

There is no consistent scientific evidence showing that screening with PSA test reduces mortality^21^ from prostate cancer. Screening actions produce short-term effects on incidence rates by detecting tumors that would not have been clinically diagnosed or that would not lead to death^19^. In the United States, where screening for prostate cancer was introduced in the 1990s, there was a decline in mortality at the beginning of the same decade^16^; however, some authors say that it would be early to attribute the reduction in mortality to the effects of screening^16,22^, since most of the observed decline may be due to other factors, especially improvement in treatment^16^.

Data from the Ambulatory Information System of SUS showed an increase in the number of PSA^23^ tests performed by health facilities located in the state of São Paulo. Between 2008 and 2018, there was an increase of 94% and an average number of annual exams of 1,368,695^23^. Among the residents in the state, partial data from the health information system on residence^24^ showed higher volumes in 2016 and 2018 (respectively 10,378 and 8,943 exams), while in 2014, 2015 and 2017, the volumes of the tests were lower (1,406, 2,506 and 4,372, respectively), indicating no consistent increase in the performance of tests.

Regarding early detection of the disease, there are no population-based data on the staging at the time of diagnosis of prostate tumors among residents in the state of São Paulo, which could raise hypotheses about the regional differences pointed out in the findings of this study. According to the Central Hospital Cancer Registry of São Paulo^25^, data for 64,745 invasive prostate tumors classified by the TNM system^26^ and diagnosed in State residents between 2000 and 2014 showed reduction in cases with extraprostatic extension, that is, advanced cases (stages III and IV). In the periods of 2000–2004, 2005–2009 and 2010–2014, the proportions of advanced stages were 41.7%, 30.6% and 28.7%, respectively^25^. The reduction in the proportion of stages III and IV was also observed in residents of 16 of the 17 regional networks, except for RHCN 3, where the proportions were 31.3%, 24.6% and 34.5% in these periods. Although there has been a reduction in cases diagnosed in advanced stages in almost all networks, these findings suggest the existence of other factors that would be related to the mortality differentials pointed out in this study, including access to diagnosis and oncological treatment and changes in therapeutic approaches in recent decades.

Regarding the effects of cancer treatment on mortality rates, it should be noted that mortality is an inaccurate indicator in the comparison between groups of patients with very different prognoses, because deaths occurred in a given year do not refer to deaths among patients who were diagnosed around the same period and possibly would have received similar oncological treatment. Thus, such measure is a slow answer to the effect of progress in cancer control regarding changes in diagnosis and the influence of treatment in prognosis^19^.

We also identified significant annual reductions in mortality in all age groups from age 50 onwards in the state of São Paulo. In the other regions, decreases were also observed, mainly in the group from 60 to 69 years. Considering that there is a higher proportion of ill-defined deaths in older patients^29,^ temporal analysis of mortality by age groups could be compromised if the reductions observed were the result of decline of quality in the filling out of death certificates. Although 74% of male deaths from ill-defined causes among residents in the state were concentrated in age groups from the age 50 onwards^30^, in the period from 2000 to 2015, there were low annual percentages (7.5% in 2000 and 4.9% in 2015). Among the regions, in the year 2000, the percentage of ill-defined causes ranged from 1% (RHCN 5 and 6) to 18% (RHCN 7 and 10); in 2015, the percentages were 1% to 13%, with a percentage below 10% in 13 regions^30^.

In the five less populous regional networks, no deaths from prostate cancer were recorded in any year between 2000 and 2015 for men between 50 and 59 years old. Although only 5% of deaths from prostate cancer occurred in individuals in this age group^8^ in the period between 2011 to 2015, we consider the temporal trend of mortality rates by age groups to be relevant, as it allows for the comparison of premature mortality by the disease with mortality at older ages for possible indication of priority measures in specific regions and/or age groups. We also considered that the statistical power of the analysis would be reduced by the distribution of total deaths to the state by regional networks of residence, causing fluctuation in the absolute number of deaths annually. We however consider the presentation of mortality trends by regions and age groups to be valid, as the most populous and most stable subgroups regarding death occurrence would show statistical significance and thus would indicate local aspects subject to action.

As limitations of the study, we can highlight that the absence of incidence data for joint analysis with mortality statistics did not allow differential mortality between regions and age groups to be better explored. Another limitation refers to the quality of answers regarding the underlying cause of death. Although the state of São Paulo presents adequate data, indicated by few ill-defined deaths and a tendency of progressive reduction^30^, the same is not uniform throughout the time series and among the regional networks.

The findings obtained in this study show a reduction in mortality in the most common type of cancer in men. Although reductions in mortality are still slight, they indicate progress in prostate cancer control actions. Screening actions and changes in therapeutic behaviors are factors that modify patterns of incidence and survival of prostate cancer in the population of São Paulo, resulting in changes in mortality rates from 2000 to 2015. In a context of limited incidence data, emphasis is given to the importance of using mortality statistics as a complement to the cancer morbidity profile.

More detailed epidemiological studies will be useful in identifying and understanding the factors that lead to interregional variations found, including data on access to health services. Some hypotheses suggested would explain some of these differences. It is also worth mentioning that investments in population-based cancer registries should have positive effects on the production of complete and quality data to support specific policies and actions, as well as contribute to the analysis of the occurrence of cancer over time.
