# Supplementary material for: Trends in prostate cancer mortality in the state of São Paulo, 2000 to 2015
Source: Rev Saude Publica. 2020 Aug 24;54:87. doi: 10.11606/s1518-8787.2020054001948 (PMC7446762; doi:10.11606/s1518-8787.2020054001948)
Supplement: Translation

# Translation: Tendências na mortalidade por câncer de próstata no estado de São Paulo, 2000 a 2015

**Authors:** Carolina Terra de Moraes Luizaga, Karina Braga Ribeiro, Luiz Augusto Marcondes Fonseca, José Eluf

**Affiliations:** I Brasil Fundação Oncocentro de São Paulo. Diretoria de Informação e Epidemiologia. São Paulo, SP, Brasil; II Brasil Faculdade de Ciências Médicas da Santa Casa de São Paulo. Departamento de Saúde Coletiva. São Paulo, SP, Brasil; III Brasil Universidade de São Paulo. Faculdade de Medicina. Hospital das Clínicas. Serviço de Imunologia Clínica e Alergia. São Paulo, SP, Brasil; IV Brasil Universidade de São Paulo. Faculdade de Medicina. Departamento de Medicina Preventiva. São Paulo, SP, Brasil; V Brasil Fundação Oncocentro de São Paulo. São Paulo, SP, Brasil

**Keywords:** Neoplasias da Próstata, mortalidade, Mortalidade, tendências, Distribuição por Idade, Distribuição Temporal

## Abstract

**OBJETIVO:**

Estimar a magnitude e identificar padrões de mudança na mortalidade por câncer de próstata no estado de São Paulo e nas 17 redes regionais de atenção à saúde, segundo grupos etários a partir dos 50 anos, no período de 2000 a 2015.

**MÉTODOS:**

As taxas de mortalidade ajustadas por idade (por 100 mil homens) foram calculadas pelo método direto usando a população mundial de Segi como padrão. A análise de regressão *Joinpoint* foi utilizada para calcular as variações percentuais anuais médias (AAPC), com intervalo de confiança de 95% (IC95%), por rede regional e grupo etário (50–59, 60–69, 70–79 e 80 anos ou mais).

**RESULTADOS:**

Para o estado de São Paulo, as taxas ajustadas de mortalidade foram de 15,2, 13,3 e 11,9/100 mil homens, respectivamente, nos períodos de 2000 a 2005, 2006 a 2010 e 2011 a 2015, com tendência de decréscimo significativo (AAPC = -2,10%; IC95% -2,42 – -1,79) a cada ano. Das 17 redes, 11 apresentaram reduções médias anuais significativas, que variaram entre -1,72% e -3,05%. A partir dos 50 anos, verificou-se redução mais acentuada nos grupos de 50 a 59 (AAPC = -2,33%; IC95% -3,04 – -1,62) e 60 a 69 anos (AAPC = -2,84%; IC95% -3,25 – -2,43).

**CONCLUSÕES:**

Embora as reduções na mortalidade ainda sejam discretas, indicam progresso nas ações de controle do câncer de próstata. Ações de rastreamento e mudanças nas condutas terapêuticas nas últimas décadas podem estar modificando a incidência e a sobrevida, resultando em mudanças no perfil de mortalidade. Estudos mais detalhados serão úteis na compreensão dos fatores que levam às variações inter-regionais encontradas.

## INTRODUÇÃO

Em todo o mundo, o câncer de próstata é o segundo mais incidente e a sexta principal causa de morte por câncer em homens^1^, mas as tendências da incidência e mortalidade pela doença variam nos diversos países. As variações geográficas das taxas de incidência são provavelmente decorrentes da combinação de casos prevalentes subjacentes e de diferenças nas práticas relacionadas ao rastreamento, incluindo o exame de PSA (antígeno prostático específico)^2,3^.

O Brasil apresenta grande extensão territorial e disparidades regionais, resultando em diferentes padrões de ocorrência de doenças, entre elas o câncer. Estima-se que, no país, o câncer de próstata seja o tipo de câncer mais incidente em homens, com número esperado de 68.220 casos novos da doença em 2018 e 2019. Este número corresponde a 31,7% de todos os cânceres (exceto o de pele não melanoma) e às taxas de incidência bruta e ajustada por idade de 66,1 e 66,8 casos novos/100 mil homens, respectivamente. Para o estado de São Paulo, nesse mesmo ano, estimaram-se 14.890 casos novos e uma taxa de incidência ajustada de 59,8 casos novos/100 mil homens^4^. A taxa de mortalidade por câncer de próstata (ajustada por idade), no Brasil, no período de 2006 a 2010, foi de 13,7/100 mil homens, com projeção de 12,9/100 mil para o período de 2011 a 2015. Para a região Sudeste, essas taxas foram de 12,9 e 11,3/100 mil nos respectivos períodos^5^.

No que se refere ao atendimento oncológico pelo Sistema Único de Saúde (SUS), há evidências de que o acesso ao diagnóstico e tratamento se distribui de forma heterogênea em São Paulo^6^, sugerindo a existência de padrões distintos de mortalidade por câncer. Este estudo teve como objetivo estimar a magnitude e identificar padrões de mudança na mortalidade por câncer de próstata em geral e segundo faixas etárias a partir dos 50 anos de idade, no estado de São Paulo e em cada uma de suas 17 redes regionais de atenção à saúde (RRAS), no período entre 2000 e 2015.

## MÉTODOS

Trata-se de um estudo ecológico, que analisou a série de dados no período de 2000 a 2015. Os óbitos por câncer de próstata constituíram o objeto de estudo. Selecionaram-se aqueles ocorridos entre residentes nas 17 RRAS do estado de São Paulo e no estado como um todo. A divisão do território em 17 RRAS, feita em 2012, teve como objetivo principal garantir a universalidade e integralidade da assistência à saúde a toda população paulista. As RRAS são definidas como arranjos organizativos de ações e serviços de saúde para integrar serviços e organizar sistemas e fluxos de informações, oferecendo suporte às atividades de planejamento e definição de fluxos no território^7^.

Os dados sobre óbitos foram coletados na página eletrônica do Departamento de Informática do Sistema Único de Saúde (Datasus), por meio de download dos arquivos provenientes do Sistema de Informações sobre Mortalidade do Ministério da Saúde^8^. As variáveis selecionadas foram: faixa etária, local de residência, ano e causa básica de morte classificada com o código C61 da 10ª revisão da Classificação Internacional de Doenças^9^. A população masculina residente no estado de São Paulo foi obtida por meio da tabulação de informações sobre estimativas populacionais^10^ para o período entre 2000 e 2015 na página eletrônica do Datasus. Para fazer a representação espacial das taxas de mortalidade no último período da série, foi utilizado o programa Tab para Windows (Tabwin versão 3.6 b)^11^.

Os óbitos foram agregados em grupos de idade quinquenais (de 0 a 80 anos ou mais) e decenais (de 50 a 80 anos ou mais). Calculamos as taxas de mortalidade ajustadas por idade tendo como base 100 mil homens. O ajuste por idade foi feito pelo método direto, utilizando como padrão a população mundial de Segi^12^. Para representar a magnitude da mortalidade, as taxas foram calculadas para três períodos compreendidos entre 2000 e 2015: período 1 (2000–2005), período 2 (2006–2010) e período 3 (2011–2015). Para a análise de tendência temporal, calculamos as taxas para cada ano da série e para quatro grupos etários (50–59, 60–69, 70–79 e 80 anos ou mais)

A avaliação da tendência temporal foi realizada pela análise de regressão *Joinpoint* para identificar os pontos de mudança em um período e para verificar a significância estatística das tendências das taxas ajustadas de mortalidade^13^. Estimamos as medidas síntese da análise de tendência, denominadas de variação percentual anual média (AAPC)^14^ que apontassem a direção (aumento ou redução) e a magnitude das mudanças no período de 2000 a 2015 e em faixas etárias a partir dos 50 anos, acompanhadas dos respectivos intervalos de confiança de 95% (IC95%), com o emprego do software *Joinpoint Regression Program*, versão 4.2.0.1^15^. Mantivemos a opção padrão do programa para ajuste de um modelo de erros não correlacionados, após testes da presença de autocorrelação serial indicarem que a análise apresentada é segura contra erros de interpretação.

Por se tratar de um estudo ecológico, a coleta de dados foi realizada em bancos de dados de domínio público, não havendo necessidade de submissão do projeto a um comitê de ética em pesquisa.

## RESULTADOS

No período de 2000 a 2015, ocorreram 40.631 óbitos por câncer de próstata entre homens residentes no estado de São Paulo (Tabela 1). No primeiro período (2000–2005), a taxa ajustada de mortalidade foi de 15,2 óbitos/100 mil homens. Entre as RRAS, as taxas variaram entre 12,1/100 mil (RRAS 12) e 18,3/100 mil (RRAS 4). Nos períodos subsequentes, as taxas para o estado foram de 13,3 e 11,9/100 mil em 2006–2010 e 2011–2015, respectivamente. No último período as taxas mais elevadas foram observadas nas RRAS 3 e 5 (15,1 e 14,6/100 mil) e as mais baixas, nas RRAS 10 e 12 (9,3/100 mil) (Figura 1).

**Tabela 1:** Redes regionais de atenção à saúde (RRAS) segundo departamento regionais de saúde (DRS) e regiões de saúde correspondentes, população masculina residente em 2015, número de óbitos e taxas de mortalidade por câncer de próstata ajustadas por idade entre 2000 e 2015.

RRAS	DRS (região de saúde)	População masculina residente (2015)	Número de óbitos (2000–2015)	Taxas de mortalidade ajustadas por idade (por 100 mil homens)		
				2000–2005	2006–2010	2011–2015
1	Grande São Paulo (Grande ABC)	1.327.156	2.263	15,7	13,1	12,7
2	Grande São Paulo (Guarulhos e Alto do Tietê)	1.431.263	1.908	16,2	14,8	14,0
3	Grande São Paulo (Franco da Rocha)	290.202	354	17,4	14,8	15,1
4	Grande São Paulo (Mananciais)	539.967	635	18,3	14,4	13,3
5	Grande São Paulo (Rota dos Bandeirantes)	898.382	1.241	17,7	15,4	14,6
6	Grande São Paulo (Município de São Paulo)	5.739.347	11.658	16,8	14,7	12,2
7	Baixada Santista e Registro	1.012.305	2.347	15,1	15,5	13,9
8	Sorocaba	1.225.859	2.144	14,2	13,2	11,5
9	Bauru	878.978	1.852	14,2	12,3	11,4
10	Marília	566.350	1.245	13,6	11,1	9,3
11	Presidente Prudente	385.065	861	12,4	12,0	10,3
12	Araçatuba São José do Rio Preto	1.180.992	2.346	12,1	10,0	9,3
13	Araraquara, Barretos, Franca e Ribeirão Preto	1.780.446	3.591	14,4	13,5	11,9
14	Piracicaba	764.711	1.386	13,5	11,6	11,5
15	Campinas e São João da Boa Vista (Rio Pardo, Mantiqueira, Baixa Mogiana, Oeste VII, Campinas)	2.005.472	3.543	15,1	11,8	11,1
16	Campinas (Bragança e Jundiaí)	612.182	1.136	15,4	12,3	11,4
17	Taubaté	1.222.358	2.121	14,1	13,7	13,2
**Estado de São Paulo**		**21.861.035**	**40.631**	**15,2**	**13,3**	**11,9**

Fontes: população: estimativas populacionais Rede Interagencial de Informações para a Saúde (RIPSA/Ministério da Saúde^10^; óbitos: Sistema de Informações sobre Mortalidade do Ministério da Saúde (Datasus)^8^.

**Figura 1:** Taxas de mortalidade ajustadas por idade (100 mil homens) por câncer de próstata segundo as 17 redes regionais de atenção à saúde do estado de São Paulo no período de 2011 a 2015.

No período estudado, observou-se um decréscimo estatisticamente significativo nas taxas de mortalidade por câncer de próstata no estado de São Paulo (AAPC = -2,10%; IC95% -2,42 – -1,79) (Figura 2). Em relação à distribuição regional, observou-se a mesma tendência em 11 das 17 regiões, com reduções médias anuais entre -1,72% (IC95% -2,46 – -0,98), na RRAS 13, e -3,05% (IC95% -4,95 – -1,10), na RRAS 10. Nas demais regiões, observou-se tendência de redução sem significância estatística (Tabela 2).

**Figura 2:** Tendência das taxas de mortalidade (ajustadas por idade) por câncer de próstata no estado de São Paulo no período de 2000 a 2015.

**Tabela 2:** Tendência das taxas de mortalidade (ajustadas por idade) por câncer de próstata nas 17 redes regionais de atenção à saúde (RRAS) no período de 2000 a 2015.

RRAS	AAPC	IC95%
1	**-2,08**	-2,93 – -1,22
2	-1,05	-2,15 – 0,06
3	-1,27	-3,49 – 1,00
4	**-2,77**	-4,16 – -1,36
5	**-2,41**	-4,21 – -0,59
6	**-2,96**	-3,31 – -2,60
7	-0,60	-1,60 – 0,42
8	**-1,89**	-3,48 – -0,28
9	**-1,90**	-3,20 -0,58
10	**-3,05**	-4,95 – -1,10
11	-1,26	-3,02 – 0,52
12	**-2,44**	-3,21 – -1,66
13	**-1,72**	-2,46 – -0,98
14	-1,28	-2,91 – 0,37
15	**-2,75**	-3,77 – -1,72
16	**-2,63**	-4,15 – -1,07
17	-0,52	-1,51 – 0,49

AAPC: variação percentual anual média; IC95%: intervalo de 95% de confiança

Nota: AAPC estatisticamente diferentes de zero em negrito

Entre 2000 e 2015, 99% dos óbitos por câncer de próstata ocorreram a partir dos 50 anos. As taxas de mortalidade específicas por idade mostraram grande variação e aumento do risco de morrer com o avançar da idade. Para o estado de São Paulo, no último ano da série, as taxas de mortalidade para os grupos quatro decenais compreendidos entre 50 e 80 anos ou mais foram, respectivamente, de 5,5, 34,4, 152,5 e 408,8 óbitos/100 mil homens.

A análise de tendência segundo faixas etárias indicou redução estatisticamente significativa da mortalidade em todos os grupos a partir dos 50 anos para o estado (Tabela 3), com os maiores percentuais de redução nas faixas de 50 a 59 (AAPC = -2,33; IC95% -3,04 – -1,62) e de 60 a 69 anos (AAPC = -2,84; IC95% -3,25 – -2,43).

**Tabela 3:** Tendência das taxas de mortalidade (ajustadas por idade) por câncer de próstata segundo grupos etários nas 17 redes regionais de atenção à saúde (RRAS) e no estado de São Paulo (ESP) no período de 2000 a 2015.

RRAS	AAPC (IC95%)			
	50 a 59 anos	60 a 69 anos	70 a 79 anos	80 anos ou mais
1	-1,10 (-4,56 – 2,48)	-2,23 (-4,99 – 0,62)	**-1,53 (-2,94 – -0,09)**	**-2,71 (-4,36 – -1,04)**
2	0,41 (-3,29 – 4,25)	**-4,03 (-6,85 – -1,12)**	0,29 (-1,46 – 2,07)	-0,91 (-2,92 – 1,15)
3	- *	**-5,90 (-9,62 – -2,03)**	**3,26 (0,19 – 6,41)**	-2,84 (-7,18 – 1,70)
4	- *	-3,98 (-9,30 – 1,65)	-2,79 (-7,33 – 1,97)	**-2,98 (-5,22 – -0,68)**
5	**-6,04 (-11,02 – -0,79)**	-2,67 (-5,60 – 0,34)	-1,79 (-4,08 – 0,54)	-2,33 (-4,62 – 0,02)
6	-1,92 (-4,05 – 0,25)	**-2,77 (-3,38 – -2,16)**	**-3,47 (-4,30 – -2,63)**	**-3,43 (-4,86 – -1,97)**
7	-1,58 (-7,03 – 4,18)	**-1,75 (-3,33 – -0,15)**	-0,68 (-2,21 – 0,88)	0,30 (-1,62 – 2,26)
8	1,16 (-15,42 – 20,98)	1,12 (-8,11 – 11,27)	-0,75 (-2,17 – 0,70)	-1,87 (-4,03 – 0,35)
9	-0,59 (-5,70 ;4,80)	**-2,77 (-5,05 – -0,44)**	**-1,88 (-3,59 – -0,15)**	-1,82 (-3,65 – 0,05)
10	- ^a^	**-4,44 (-7,09 – -1,72)**	-2,47 (-5,16 – 0,30)	**-2,55 (-4,75 – -0,29)**
11	- ^a^	**-3,07 (-6,04 – -0,01)**	-1,50 (-3,76 – 0,80)	0,32 (-2,04 – 2,73)
12	-2,52 (-7,23 – 2,43)	**-2,40 (-3,29 – -1,50)**	**-3,24 (-4,31 – -2,16)**	-1,42 (-3,01 – 0,19)
13	-0,51 (-3,20 – 2,25)	**-3,65 (-5,23 – -2,05)**	**-0,99 (-1,96 – -0,01)**	**-1,58 (-2,94 – -0,20)**
14	- *	-1,30 (-4,31 – 1,81)	-1,18 (-3,35 – 1,03)	-1,83 (-4,38 – 0,79)
15	**-4,79 (-6,99 – -2,55)**	**-3,44 (-5,23 – -1,61)**	**-2,02 (-3,40 – -0,62)**	**-2,74 (-4,14 – -1,32)**
16	-4,40 (-8,61 – 0,00)	-2,18 (-5,00 – 0,73)	-2,12 (-4,64 – 0,47)	**-2,95 (-4,79 – -1,08)**
17	-4,41 (-9,08 – 0,49)	**-3,93 (-6,15 – -1,65)**	0,26 (-1,30 – 1,84)	0,57 (-1,55 – 2,73)
ESP	**-2,33 (-3,04 – -1,62)**	**-2,84 (-3,25 – -2,43)**	**-1,93 (-2,36 – -1,50)**	**-1,92 (-2,40 – -1,44)**

AAPC: variação percentual anual média; IC95%: intervalo de 95% de confiança

Nota: AAPC estatisticamente diferentes de zero em negrito

* A AAPC não foi calculada pois não houve registro de óbito por câncer de próstata em pelo menos um ano da série entre 2000 e 2015.

Na análise estratificada por regiões, no grupo de 50 a 59 anos, observaram-se reduções significativas na RRAS 5 (AAPC = -6,04; IC95% -11,02 – -0,79) e na RRAS 15 (AAPC = -4,79; IC95% -6,99 – -2,55). No grupo de 60 a 69 anos, em 11 das 17 RRAS, verificaram-se reduções significativas na mortalidade, principalmente na RRAS 2 e na RRAS 3. No grupo de 70 a 79 anos, o maior declínio (AAPC = -3,47; IC95% -4,30 – -2,63) foi observado na RRAS 6 e reduções significativas, de pelo menos 0,99%, em outras cinco RRAS. Na faixa de 80 anos ou mais, decréscimos significativos foram observados em sete regiões, sendo que a RRAS 6 apresentou a redução de maior magnitude (AAPC = -3,43; IC95% -4,86 – -1,97) (Tabela 3).

## DISCUSSÃO

No período de 2000 a 2015, houve um decréscimo estatisticamente significativo das taxas de mortalidade por câncer de próstata no estado de São Paulo e em 11 das 17 RRAS. Mundialmente a tendência temporal de redução da mortalidade por esse tipo de câncer foi encontrada em áreas desenvolvidas como Reino Unido (-1,14% a cada ano entre 1992 e 2004) e Estados Unidos (-4,17% entre 1994 e 2004^16^ e -7,19% entre 2009 e 2013)^17^. Para o Brasil e suas regiões, entre 1980 e 2010, observou-se tendência ascendente em um estudo que incluiu a redistribuição de óbitos por causas mal definidas na correção das taxas de mortalidade^18^.

No estado de São Paulo, no período de 2011 a 2015 a taxa de mortalidade por câncer de próstata ajustada por idade (11,9 óbitos/100 mil homens) foi inferior ao risco de morte estimado para a América do Sul em 2018^1^ (14/100 mil), superior ao estimado para América do Norte, Sul da Europa e Ásia Ocidental (8/100 mil), e similar ao estimado para Reino Unido (13/100 mil), Colômbia e Argentina (12/100 mil)^1^. Globalmente as taxas de mortalidade por câncer de próstata apresentam menor variabilidade geográfica do que a incidência da doença^1^. Os maiores riscos de morte são observados em regiões menos desenvolvidas e onde há predomínio de população negra, como nas regiões do Caribe e África Subsaariana (taxas de 23 a 27/100 mil)^1^. Quanto à tendência temporal, declínios foram notados previamente em áreas de recursos mais elevados e aumento em países de poucos recursos^2^.

Estudos de mortalidade são essenciais para indicar prioridades em saúde pública; entretanto, as interpretações a partir dessa medida isolada podem ser falaciosas. Tendências da mortalidade por câncer são resultado de tendências anteriores tanto da incidência como da sobrevida^19^. No estado de São Paulo, assim como no Brasil, a incidência de câncer está disponível para poucos municípios cobertos por registros de câncer de base populacional (RCBP), ou é produzida por meio de estimativas para estados e capitais pelo Instituto Nacional do Câncer desde 1995^4^. Por questões metodológicas, essas estimativas não devem ser utilizadas para estudos de séries temporais. Apesar de existirem cinco RCBP em atividade no estado, que fornecem dados de incidência para seus municípios de cobertura^20^, não foi possível neste estudo avaliar os efeitos das taxas de incidência e sobrevida do câncer de próstata na mortalidade pela doença, seja pela pequena representatividade populacional no âmbito estadual, seja pela instabilidade nas taxas ao longo do tempo.

Não há evidências científicas consistentes demonstrando que o rastreamento com teste do PSA reduza a mortalidade^21^ pelo câncer de próstata. Ações de rastreamento produzem efeitos de curto prazo nas taxas de incidência pela detecção de tumores que não teriam sido diagnosticados clinicamente ou que não causariam mortes^19^. Nos Estados Unidos, onde o rastreamento para o câncer de próstata foi introduzido nos anos 1990, observou-se declínio da mortalidade no início da mesma década^16^; no entanto, alguns autores afirmam que seria precoce atribuir a redução da mortalidade aos efeitos do rastreamento^16,22^, pois a maior parte do declínio observado pode ser devida a outros fatores, especialmente à melhora no tratamento^16^.

Dados do Sistema de Informações Ambulatoriais do SUS mostraram aumento no número de exames de PSA^23^ realizados por estabelecimentos de saúde localizados no estado de São Paulo. Entre 2008 e 2018, verificou-se incremento de 94% e número médio de exames anuais de 1.368.695^23^. Entre os residentes no estado, dados parciais do sistema de informação em saúde que dispunha da informação sobre o local de residência^24^ mostraram maiores volumes em 2016 e 2018 (respectivamente de 10.378 e 8.943 exames), ao passo que em 2014, 2015 e 2017, os volumes dos exames foram mais baixos (1.406, 2.506 e 4.372, respectivamente), não indicando aumento consistente na realização de exames.

Acerca da detecção precoce da doença, não existem dados de base populacional sobre o estadiamento no momento do diagnóstico dos tumores de próstata entre residentes no estado de São Paulo, os quais poderiam suscitar hipóteses sobre as diferenças regionais apontadas nos achados deste estudo. Segundo o Registro Hospitalar de Câncer de São Paulo^25^, dados de 64.745 tumores invasivos de próstata estadiáveis pela classificação TNM^26^ diagnosticados entre residentes no estado entre 2000 e 2014 mostraram redução na proporção de casos com extensão extraprostática ao diagnóstico, isto é, casos em estádios avançados (III e IV). Nos períodos de 2000–2004, 2005–2009 e 2010–2014, as proporções de estádios avançados foram, respectivamente, de 41,7%, 30,6% e 28,7%^25^. A redução na proporção de estádios III e IV também foi observada em residentes de 16 das 17 RRAS, exceto pela RRAS 3, onde as proporções foram 31,3%, 24,6% e 34,5% nos referidos períodos. Embora tenha havido redução na proporção de casos diagnosticados em estádios avançados em quase todas as RRAS, esses achados sugerem a existência de outros fatores que estariam relacionados aos diferenciais de mortalidade apontados neste estudo, entre eles o acesso ao diagnóstico e tratamento oncológico e mudanças nas condutas terapêuticas nas últimas décadas.

Em relação aos efeitos do tratamento oncológico sobre as taxas de mortalidade, é de se destacar que a mortalidade constitui-se como indicador pouco preciso na comparação entre grupos de pacientes com prognósticos muito diferentes, pois as mortes ocorridas em um determinado ano não se referem às mortes entre pacientes que foram diagnosticados em torno do mesmo período e, possivelmente, teriam recebido tratamento oncológico semelhante. Assim, essa medida reflete tardiamente o efeito de progressos no controle do câncer quando se trata de avaliar como as mudanças no diagnóstico e tratamento influenciam o prognóstico^19^.

Identificamos ainda reduções anuais significativas na mortalidade em todos os grupos etários a partir dos 50 anos no estado de São Paulo. Nas demais regiões decréscimos também foram observados, principalmente no grupo de 60 a 69 anos. Considerando que há elevada proporção de causas mal definidas nas idades avançadas^29^, a análise temporal da mortalidade por grupos de idade poderia estar comprometida se as reduções observadas fossem resultantes de piora na qualidade do preenchimento das declarações de óbito. Apesar de 74% dos óbitos masculinos por causas mal definidas entre residentes no estado estarem concentrados em grupos etários a partir dos 50 anos^30^, no período de 2000 a 2015, foram verificados baixos percentuais anuais (7,5% em 2000 e 4,9% em 2015). Entre as regiões, em 2000, o percentual de causas mal definidas variou de 1% (RRAS 5 e 6) a 18% (RRAS 7 e 10); em 2015, os percentuais foram de 1% a 13%, com percentual inferior a 10% em 13 regiões^30^.

Nas cinco RRAS menos populosas, no grupo de homens entre 50 e 59 anos, não foram registrados óbitos por câncer de próstata em algum ano entre 2000 e 2015. Embora apenas 5% dos óbitos por câncer de próstata tenham ocorrido em indivíduos nessa faixa etária^8^ no período de 2011 a 2015, consideramos relevante apresentar a tendência temporal das taxas de mortalidade por grupos de idade, permitindo comparar a mortalidade prematura pela doença com a mortalidade em idades mais avançadas para possível indicação de medidas prioritárias em regiões e/ou grupos etários específicos. Ponderamos também que o poder estatístico da análise estaria reduzido pela distribuição dos óbitos totais para o estado por RRAS de residência, ocasionando flutuação no número absoluto de mortes ano a ano. Entretanto, consideramos válido apresentar a tendência da mortalidade por regiões e grupos etários, pois os subgrupos mais populosos e com maior estabilidade na ocorrência das mortes mostrariam significância estatística e indicariam aspectos locais passíveis de ações.

Como limitações do estudo, podemos destacar que a ausência de dados de base populacional para análise conjunta com as estatísticas de mortalidade não permitiu que a mortalidade diferencial entre regiões e grupos etários fosse melhor explorada. Outra limitação refere-se à qualidade do preenchimento da causa básica de morte. Embora o estado de São Paulo apresente adequada validade dos dados, indicada pelas baixas proporções de óbitos por causas mal definidas e tendência de redução progressiva ao longo dos anos^30^, pode-se considerar que a qualidade de preenchimento das declarações de óbito não é uniforme ao longo da série temporal e também entre as RRAS.

Os achados obtidos neste estudo evidenciam a redução na mortalidade pelo tipo de câncer mais incidente em homens. Embora as reduções observadas ainda sejam discretas, indicam progresso nas ações de controle do câncer de próstata na população do estado de São Paulo. Ações de rastreamento e mudanças nas condutas terapêuticas nas últimas décadas constituem fatores que estariam modificando padrões de incidência e de sobrevida pelo câncer de próstata na população paulista, resultando nas mudanças nas taxas de mortalidade no período de 2000 a 2015. Em um contexto de limitada disponibilidade de dados sobre incidência, ressalta-se a importância da utilização das estatísticas de mortalidade como complemento do perfil de morbidade por câncer.

Estudos epidemiológicos mais detalhados serão úteis na identificação e compreensão dos fatores que levam às variações inter-regionais encontradas, incluindo dados de acesso aos serviços de saúde. Algumas hipóteses aventadas explicariam parte dessas diferenças. Cabe ainda ressaltar que investimentos em registros de câncer de base populacional devem surtir efeitos positivos na produção de dados completos e de qualidade para subsidiar políticas e ações específicas, bem como contribuir para a análise da ocorrência do câncer ao longo do tempo.
